# Prevalence and diagnostic reclassification of sarcopenia in type 2 diabetes according to AWGS 2019 and AWGS 2025 criteria: a secondary cross-sectional analysis

**DOI:** 10.1186/s12902-026-02428-2

**Published:** 2026-08-13

**Authors:** Pitchaporn Phudphong, Kwanchanok Laoticharoen, Nath Adulkasem

**Affiliations:** 1 Division of Endocrinology and Metabolism, Department of Medicine, Faculty of Medicine, Vajira Hospital, Navamindradhiraj University, Dusit, Bangkok, 10300 Thailand; 2 Department of Orthopedics Surgery, Faculty of Medicine Siriraj Hospital, Mahidol University, Bangkok, 10700 Thailand

**Keywords:** Sarcopenia, Type 2 diabetes mellitus, Asian Working Group for Sarcopenia (AWGS), Body mass index, Diagnostic criteria

## Abstract

**Background:**

Sarcopenia is common in patients with type 2 diabetes mellitus (T2DM). In 2025, the Asian Working Group for Sarcopenia (AWGS) updated its diagnostic criteria by removing physical performance from the diagnostic algorithm and introducing BMI-adjusted appendicular skeletal muscle mass (ASM). These changes may influence sarcopenia detection in populations with altered body composition, including those with T2DM. This study compared sarcopenia prevalence and diagnostic transitions between AWGS 2019 and AWGS 2025 in patients with T2DM.

**Methods:**

This study represents a secondary analysis of an existing cross-sectional dataset collected between February and November 2025. Participants were adults aged 40–90 years with T2DM treated for at least one year. Body composition was assessed using multi-frequency bioelectrical impedance analysis, and handgrip strength and physical performance were evaluated using standardized protocols. Sarcopenia was defined using the AWGS 2019 and 2025 criteria. Prevalence, clinical characteristics, and diagnostic transitions were compared.

**Results:**

Among 250 participants (mean age 65.1 ± 8.5 years; mean BMI 27.9 ± 5.5 kg/m²), sarcopenia prevalence was substantially higher using the AWGS 2025 than the AWGS 2019 criteria (48.4%, 95% CI 42.2–54.6 vs. 23.2%, 95% CI 18.4–28.9; McNemar *p* < 0.001). Overall agreement between the two classifications was 66.0% (Cohen’s κ = 0.31). Increases were observed across most age groups. Patients classified as sarcopenic according to the AWGS 2025 criteria had higher BMI, consistent with the incorporation of BMI-adjusted ASM in the revised criteria, as well as more frequent insulin use and higher uACR. Diagnostic transitions showed that 74 patients (29.6%) met the AWGS 2025 criteria for sarcopenia but not the AWGS 2019 criteria.

**Conclusions:**

The AWGS 2025 criteria classified substantially more patients with T2DM as having sarcopenia than the AWGS 2019 criteria. Participants additionally identified under the AWGS 2025 criteria exhibited distinct metabolic characteristics, including more frequent insulin use and elevated uACR. Further longitudinal studies are needed to determine the clinical relevance of these diagnostic reclassifications.

**Trial registration:**

Not applicable. This study is a secondary analysis of previously collected cross-sectional data and does not involve any health care intervention.

**Supplementary Information:**

The online version contains supplementary material available at https://doi.org/10.1186/s12902-026-02428-2.

## Background

Sarcopenia is increasingly recognized as an important complication of type 2 diabetes mellitus (T2DM), with individuals with diabetes having an approximately 1.5–2-fold higher risk than those without diabetes [1]. Beyond age-related muscle decline, T2DM contributes to accelerated deterioration in muscle health through chronic hyperglycemia, insulin resistance, inflammation, oxidative stress, and accumulation of advanced glycation end products (AGEs), all of which impair muscle structure and function [2–6]. As a result, sarcopenia in T2DM is associated with impaired physical performance, functional decline, and poorer clinical outcomes [7].

A distinctive feature of T2DM is its altered body composition. Many patients are overweight or obese, with increased adiposity while simultaneously exhibiting reduced muscle mass and muscle quality [8]. Consequently, absolute or height-adjusted appendicular skeletal muscle mass (ASM) may appear preserved despite clinically meaningful relative muscle deficiency. This phenotype, particularly common among individuals with obesity or sarcopenic obesity, may lead to underrecognition of sarcopenia when conventional height-adjusted ASM is used [9, 10].

The reported prevalence of sarcopenia varies considerably across studies owing to differences in participant characteristics, disease severity, sex distribution, and, importantly, the diagnostic criteria applied. As the definition of sarcopenia has evolved, prevalence estimates have changed accordingly. For example, in some study populations, the Asian Working Group for Sarcopenia (AWGS) criteria have identified a higher prevalence of sarcopenia than the European Working Group on Sarcopenia in Older People (EWGSOP) or the Foundation for the National Institutes of Health (FNIH) criteria, reflecting differences in cut-off values and approaches to muscle assessment [11]. Similarly, the AWGS 2019 definition has been reported to yield a higher prevalence than AWGS 2014 [12]. These findings suggest that the choice of diagnostic criteria can substantially influence prevalence estimates, case identification, the timing of intervention, and healthcare planning.

In 2025, AWGS introduced updated diagnostic criteria for Asian populations, incorporating several modifications to the diagnostic framework. Key changes include lowering cut-off values for middle-aged adults, removing physical performance from the diagnostic algorithm, and adding a BMI-adjusted appendicular skeletal muscle mass criterion. This modification accounts for BMI and may improve recognition of relative muscle deficiency, particularly among individuals with obesity or sarcopenic obesity [9]. Conversely, it may increase the likelihood of overclassifying individuals with obesity as sarcopenic because BMI does not distinguish fat mass from lean mass. Consequently, the revised criteria may substantially alter the estimated prevalence and diagnostic classification of sarcopenia, particularly in populations with a high prevalence of obesity. Given that T2DM is commonly associated with elevated BMI and altered body composition, the updated criteria may substantially affect sarcopenia prevalence in this population [8]. Because the AWGS 2025 criteria were only recently introduced, evidence regarding their effects on sarcopenia prevalence and diagnostic reclassification remains limited, particularly among individuals with T2DM. Therefore, understanding how the revised criteria influence sarcopenia prevalence and classification in individuals with T2DM is important.

This study aimed to compare the prevalence of sarcopenia according to the AWGS 2019 and AWGS 2025 criteria among the same cohort of adults with T2DM. We also characterized the clinical profiles of participants classified by each definition and examined diagnostic reclassification to describe the characteristics of individuals whose classification differed between the two criteria. These findings provide insight into the impact of the revised AWGS 2025 criteria on sarcopenia prevalence and diagnostic classification in adults with T2DM. This study was designed to describe changes in prevalence and diagnostic reclassification rather than to determine whether either definition more accurately predicts clinically relevant outcomes.

## Methods

### Study design and participants

This study is a secondary analysis of previously collected cross-sectional data obtained between February and November 2025 to assess muscle mass, strength, and physical performance in patients with T2DM. The parent study was a single-center cross-sectional study conducted at a university-affiliated tertiary medical center to investigate the prevalence and risk factors of sarcopenia and fracture risk among adults with type 2 diabetes mellitus (T2DM).

Consecutive eligible adults aged 40–90 years attending the outpatient diabetes clinic who had been diagnosed with and treated for T2DM for at least one year were recruited. The parent study included adults aged 40 years and older to capture the full spectrum of T2DM, given the evidence that muscle impairment may occur earlier in individuals with T2DM than in the general population. Participants were excluded if they had conditions known to independently affect muscle metabolism, body composition, or physical performance. Advanced chronic kidney disease, chronic liver disease, gastrointestinal malabsorption, active malignancy, and autoimmune or rheumatologic disorders were excluded because these conditions are recognized causes of secondary sarcopenia and may alter muscle mass independently of T2DM [9, 13]. Participants with a history of fragility fracture, osteoporosis, or ongoing osteoporosis treatment were excluded to minimize confounding related to bone-muscle interactions. Individuals considered unsafe for physical performance testing were excluded to ensure participant safety and the validity of functional assessments. Finally, participants with contraindications to bioelectrical impedance analysis, including pregnancy or implanted cardiac electronic devices, were excluded because these conditions may compromise the safety or accuracy of body composition measurements [9, 14, 15].

**Fig. 1:**
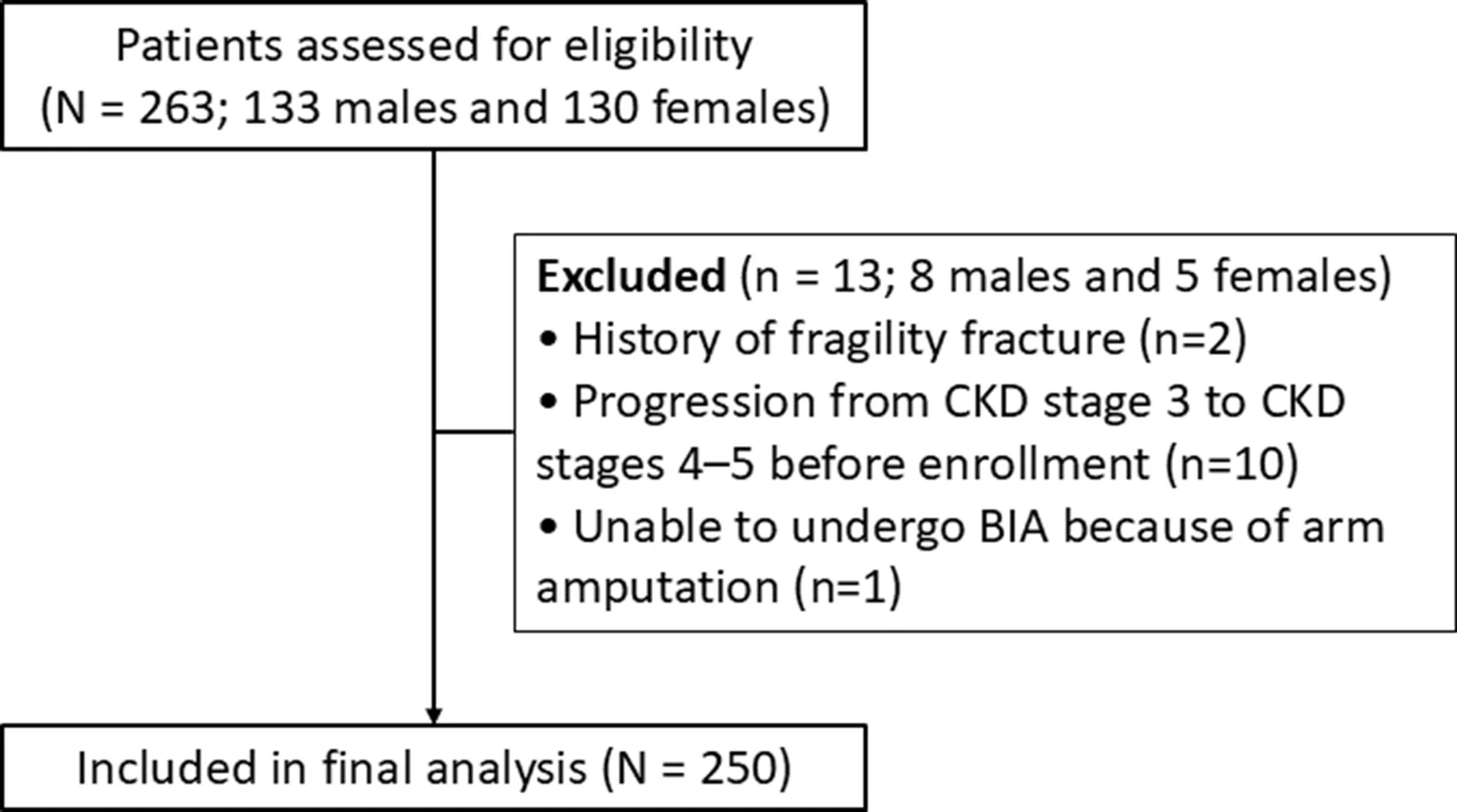
Flow diagram of participant eligibility and inclusion

Of the 263 patients assessed for eligibility, 13 were excluded: 10 because their chronic kidney disease progressed from stage 3 to stages G4–G5 before enrollment, two because of a history of fragility fracture, and one because BIA could not be performed due to unilateral arm amputation. Consequently, 250 participants were included in the final analysis (Fig. 1).

All eligible participants with complete data were included in the analysis to maximize the precision of the estimates. As this was a secondary analysis of an existing dataset, no dedicated sample size calculation was performed. The parent study protocol was approved by the institutional review board, and written informed consent was obtained from all participants during the original data collection. As the present secondary analysis was not prespecified in the original study protocol, it underwent independent ethical review and approval under a separate institutional review board protocol. All procedures performed in this study involving human participants were conducted in accordance with the ethical standards of the institutional and national research committee and with the principles of the Declaration of Helsinki.

### Data sources and clinical covariates

Eligible participants attended a single study visit during which all study-related assessments and data collection were performed. Demographic and clinical characteristics were obtained, including age, sex, body mass index (BMI), anthropometric measurements (neck, waist, mid-upper arm, and calf circumferences), smoking status, alcohol intake, physical activity, comorbid conditions, and T2DM-related information such as duration of diabetes, medication use, and diabetes-related complications.

Physical activity was assessed by self-report and categorized according to the average duration of planned exercise during the preceding 3 months as none, < 150 min/week, or ≥ 150 min/week. Comorbid conditions, including hypertension, dyslipidemia, and established cardiovascular disease, were identified from physician-documented medical records and current treatment. Diabetes-related complications included diabetic retinopathy, peripheral neuropathy, and albuminuria. Albuminuria was categorized according to the urine albumin-to-creatinine ratio (uACR) as < 30, 30–300, or > 300 mg/g based on routine clinical assessment and medical records. Medication use was obtained from the medication list at the study visit. Participants were categorized as receiving insulin therapy or no insulin therapy. Among insulin users, the total daily insulin dose was calculated from the prescribed daily dose and expressed as units/kg/day. Insulin dose was further categorized as low (< 0.5 units/kg/day), moderate (0.5–1.0 units/kg/day), or high (> 1.0 units/kg/day).

Routine laboratory parameters, including fasting plasma glucose, glycated hemoglobin (HbA1c), lipid profile, complete blood count, renal function tests, liver function tests, and uACR, were obtained either on the study visit date or within 7 days before the scheduled clinic visit according to institutional practice. For selected laboratory tests that were not routinely repeated at every visit, such as serum 25-hydroxyvitamin D (25(OH)D), the most recent available result obtained within the preceding 3 months was used when repeat testing was not clinically indicated. Complete data were available for all laboratory variables included in the analysis.

### Anthropometric and body composition measurements

#### Anthropometric Measurements

Anthropometric parameters were evaluated using standardized measurement techniques. Neck circumference (NC) was measured while participants stood in an upright position with the head aligned in the Frankfort horizontal plane. A flexible non-stretch tape was applied around the neck just below the thyroid cartilage and kept perpendicular to the neck axis without exerting pressure on the skin [16].

Waist circumference (WC) was determined in accordance with World Health Organization recommendations. Measurements were obtained at the midpoint between the lowest rib and the iliac crest using a horizontal non-elastic tape after normal expiration while the participant remained standing [17].

Mid-upper arm circumference (MUAC) was assessed on the non-dominant arm with the arm relaxed naturally beside the body. The midpoint between the acromion and olecranon landmarks was identified, and circumference was measured with a non-stretch tape to the nearest 0.1 cm [18].

Calf circumference (CC) was recorded from the leg with the greater circumference while participants were standing. Measurements were obtained using a flexible non-elastic tape positioned horizontally around the widest part of the calf without compressing the soft tissue, and values were documented to the nearest 0.1 cm [9, 19].

### Muscle health assessments

#### Muscle mass evaluation

We evaluated body composition using a multi-frequency BIA (InBody 270, InBody Co., Ltd., Seoul, Korea). Measurements included appendicular skeletal muscle mass (ASM), body fat percentage, and basal metabolic rate (BMR). All assessments were performed in the morning before noon, after an overnight fast of at least 8 h, and following bladder voiding, in accordance with the manufacturer’s recommendations and standardized BIA protocols [15]. Participants were also instructed to avoid vigorous exercise and excessive fluid intake before assessment, and were measured barefoot. Peripheral edema was assessed before measurement, and participants with clinically evident edema were noted. ASM was obtained directly from the InBody device using the manufacturer’s proprietary algorithm. Height-adjusted ASM (ASMI) was calculated as ASM divided by height squared (kg/m²), and BMI-adjusted ASM was calculated as ASM divided by BMI, in accordance with the AWGS 2025 recommendations [9, 14].

#### Muscle strength evaluation

We assessed muscle strength using a Smedley-type handgrip dynamometer (Takei 5401 Digital Dynamometer; Takei, Tokyo, Japan). Participants performed two maximal-effort trials while standing with their dominant arm fully extended at their side [20]. The higher of the two values was used for analysis.

#### Physical performance evaluation

We evaluated physical performance using two tests, gait speed and the five-times chair stand (FTCS) test. For gait speed, participants were asked to walk at their usual pace over a six-meter distance for two trials [14]. The average time was used to calculate gait speed. For the FTCS test, participants were instructed to perform five consecutive sit-to-stand movements as quickly as possible from a standard chair with a backrest, keeping their arms crossed [21]. The time required to complete all five repetitions was recorded.

### Sarcopenia definition

Sarcopenia was diagnosed using the AWGS 2019 and AWGS 2025 definitions [9, 14]. In the 2019 criteria, sarcopenia is defined by reduced skeletal muscle mass measured using height-adjusted ASM, together with either low handgrip strength, low physical performance, or both [14]. In the updated 2025 version, sarcopenia is defined by reduced muscle mass and reduced handgrip strength, without requiring impaired physical performance [9]. Muscle mass can be classified as low using either height-adjusted ASM or BMI-adjusted ASM. Participants younger than 50 years were classified using the AWGS 2025 cutoffs for the 50–65-year age group, as this was the closest applicable age category. A sensitivity analysis excluding these participants was performed to evaluate the impact of their inclusion on the study findings. The detailed diagnostic definitions used in this study are provided in the Supplementary Table 1.

### Diagnostic reclassification

Concordant sarcopenia was defined as participants meeting diagnostic criteria for sarcopenia under both AWGS 2019 and AWGS 2025 definitions, whereas concordant non-sarcopenia referred to participants classified as non-sarcopenic by both criteria.

Reclassified sarcopenia referred to participants who were categorized as non-sarcopenic under AWGS 2019 but met the diagnostic criteria for sarcopenia according to AWGS 2025. Reclassified non-sarcopenia referred to participants who fulfilled sarcopenia criteria under AWGS 2019 but were subsequently classified as non-sarcopenic using AWGS 2025.

### Statistical analysis

Statistical analyses were performed using Stata version 16 (StataCorp, College Station, TX). Continuous variables were summarized as mean ± SD or median (IQR) and compared using t-tests or Mann–Whitney U tests as appropriate. Categorical variables were presented as frequencies and percentages and compared using chi-square or Fisher’s exact tests. A two-sided p-value < 0.05 was considered statistically significant.

The prevalence of sarcopenia under each criterion, stratified by age group, was illustrated using bar charts. Prevalence under each criterion was reported with 95% confidence intervals. The paired difference in prevalence between the two criteria was tested with McNemar’s test, and agreement between the two classifications was quantified using overall percent agreement and Cohen’s kappa. Characteristics across the four diagnostic transition groups were compared using one-way ANOVA with Bonferroni adjustment for multiple comparisons; categorical variables were compared using the chi-square or Fisher’s exact test. We performed exploratory descriptive comparisons between sarcopenic and non-sarcopenic participants under the AWGS 2019 and AWGS 2025 definitions. These analyses were intended to describe the characteristics of the populations identified by each definition rather than to test prespecified hypotheses. Diagnostic transitions between the two criteria were assessed by identifying participants with concordant and reclassified diagnoses, which were visualized using a Sankey diagram. We examined diagnostic transitions by comparing concordant classifications with patients whose diagnoses changed between AWGS 2019 and AWGS 2025.

## Results

**Fig. 2:**
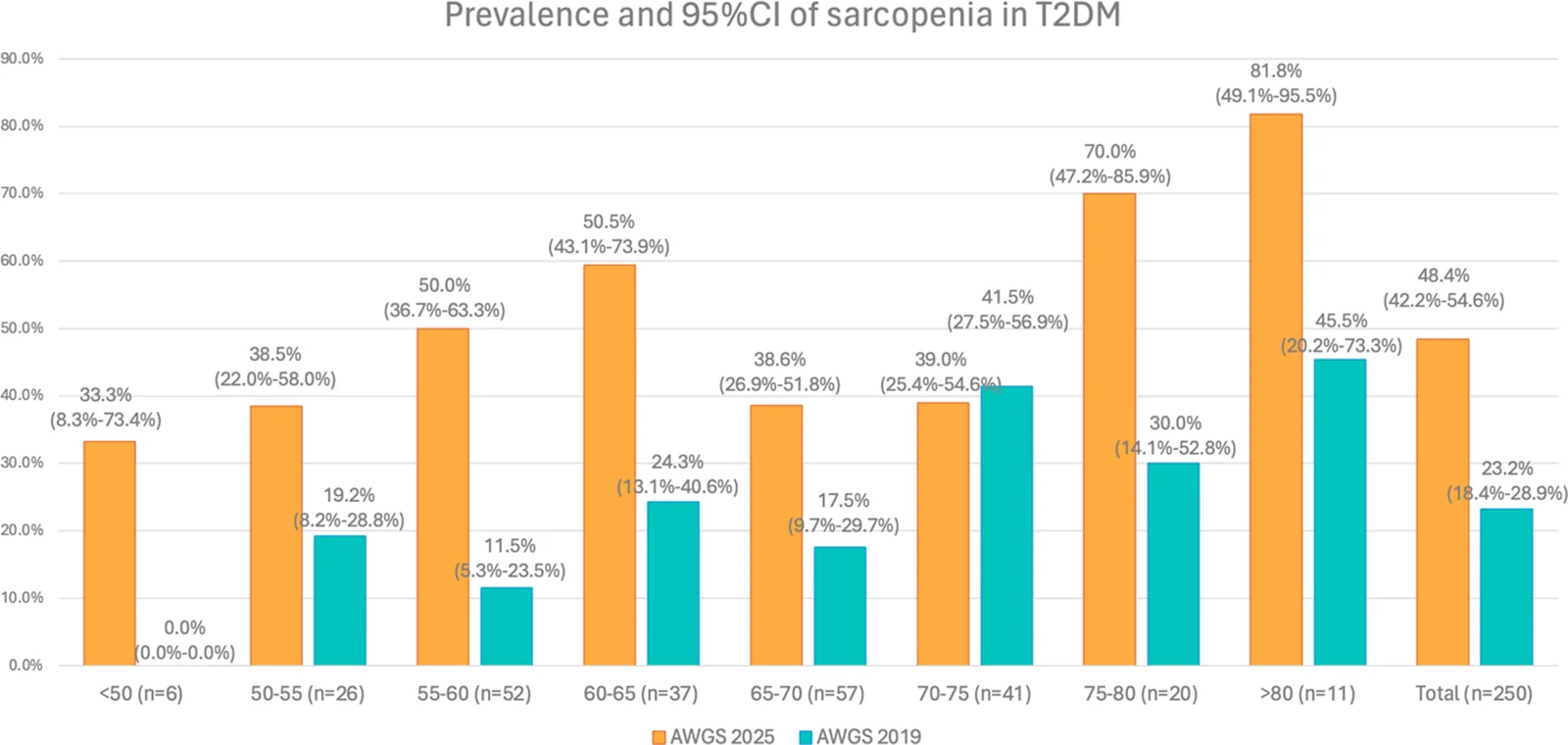
Prevalence of sarcopenia in type 2 diabetes mellitus patients across age groups according to the AWGS 2019, and 2025 criteria

A total of 250 patients with T2DM were included. The mean age was 65.1 ± 8.5 years, and the average BMI was 27.9 ± 5.5 kg/m². Glycemic control was generally adequate, with a mean HbA1c of 7.7 ± 1.5%. Overall, the prevalence of sarcopenia was markedly higher using the AWGS 2025 criteria than the AWGS 2019 criteria (48.4%, 95% CI 42.2–54.6 vs. 23.2%, 95% CI 18.4–28.9), representing an absolute difference of 25.2% points (95% CI 17.1–33.3; McNemar *p* < 0.001) (Fig. 2**). **Six participants were younger than 50 years. Sensitivity analysis excluding the six participants aged < 50 years yielded similar prevalence estimates of 23.8% (95% CI 18.8–29.5%) for the AWGS 2019 definition and 48.8% (95% CI 42.5–55.1%) for the AWGS 2025 definition. Higher prevalence under AWGS 2025 was observed across almost all age groups, showing a bimodal pattern with peaks in the early elderly (60–65 years) and the oldest group (> 80 years).

Across both AWGS versions, sarcopenic patients tended to be older, with the 2025 criteria identifying a slightly younger group (Table 1). There was no significant difference in sex distribution. BMI patterns differed between the two criteria. AWGS 2025 identified sarcopenic T2DM patients with higher BMI than AWGS 2019, resulting in a more balanced BMI distribution between the sarcopenia and non-sarcopenia groups. Patients classified as sarcopenic under AWGS 2025 demonstrated different metabolic characteristics. Among insulin users, the direction of the difference in mean insulin dose was reversed under the two definitions. Under the AWGS 2019 definition, sarcopenic participants had a lower mean insulin dose than non-sarcopenic participants, whereas under the AWGS 2025 definition, they had a higher mean insulin dose. A similar descriptive pattern was observed for the uACR. Nevertheless, both criteria demonstrated comparable ability to distinguish sarcopenic from non-sarcopenic patients in terms of appendicular skeletal muscle mass, muscle strength, and physical performance.

**Table 1:** Characteristics and functional outcomes of patients with sarcopenia according to AWGS 2025 and AWGS 2019 criteria

Characteristics	AWGS 2025			AWGS 2019		
	Sarcopenia *n* = 121 (48.4%)	Non-sarcopenia *n* = 129 (51.6%)	*p*-value	Sarcopenia *n* = 58 (23.2%)	Non-sarcopenia *n* = 192 (76.8%)	*p*-value
Age (years)	66.2 ± 9.0	64.1 ± 7.9	**0.049**	68.6 ± 8.7	64.1 ± 8.2	**< 0.001**
Sex, n (%)						
Male	64 (52.9%)	61 (47.3%)	0.448	31 (53.5%)	94 (49.0%)	0.653
Female	57 (47.1%)	68 (52.7%)		27 (46.6%)	98 (51.0%)	
Weight (kg)	68.0 ± 14.5	75.0 ± 17.1	**< 0.001**	57.3 ± 9.5	75.9 ± 15.3	**< 0.001**
Height (cm)	157.6 ± 7.2	161.5 ± 8.2	**< 0.001**	156.0 ± 7.6	160.7 ± 7.8	**< 0.001**
BMI^1^ (kg/m^2^)	27.3 ± 5.3	28.6 ± 5.6	0.056	23.4 ± 3.8	29.3 ± 5.2	**< 0.001**
T2DM^2^ duration (years)	17.0 ± 9.8	15.0 ± 9.5	0.117	18.2 ± 10.6	15.3 ± 9.3	**0.046**
HbA1c (%)	7.7 ± 1.5	7.6 ± 1.6	0.606	7.7 ± 1.6	7.6 ± 1.5	0.739
Insulin use, n (%)						
No insulin therapy	64 (52.9%)	74 (57.4%)	0.226	39 (67.2%)	99 (51.6%)	**0.030**
Low dose (< 0.5 unit/kg/day)	27 (22.3%)	36 (27.9%)		15 (25.9%)	48 (25.0%)	
Moderate dose (0.5-1.0 unit/kg/day)	24 (19.8%)	16 (12.4%)		4 (6.9%)	36 (18.8%)	
High dose (> 1.0 unit/kg/day)	6 (5.0%)	3 (2.3%)		0 (0.0%)	9 (4.7%)	
Total insulin use (unit/kg/day)^†^	0.55 ± 0.32	0.44 ± 0.28	**0.048**	0.36 ± 0.23	0.52 ± 0.31	**0.036**
uACR^3^, n (%)						
<30 mg/g	57 (47.1%)	84 (65.1%)	**0.013**	31 (53.5%)	110 (57.3%)	0.726
30–300 mg/g	46 (38.0%)	35 (27.1%)		19 (32.8%)	62 (32.3%)	
>300 mg/g	18 (14.9%)	10 (7.8%)		8 (13.8%)	20 (10.4%)	
BMR^4^ (kcal/day)	1308.0 ± 165.9	1418.1 ± 243.6	**< 0.001**	1207.8 ± 123.3	1412.2 ± 216.3	**< 0.001**
Body Fat (%)	34.9 ± 9.3	35.4 ± 8.8	0.672	31.2 ± 9.5	36.4 ± 8.6	**< 0.001**
ASM^5^ (kg)	16.9 ± 3.9	19.5 ± 4.8	**< 0.001**	14.4 ± 2.9	19.4 ± 4.3	**< 0.001**
ASM/h^2^ (kg/m^2^)	6.8 ± 1.1	7.4 ± 1.2	**< 0.001**	5.9 ± 0.7	7.4 ± 1.1	**< 0.001**
ASM/BMI	0.6 ± 0.2	0.7 ± 0.2	**0.010**	0.6 ± 0.1	0.7 ± 0.2	**0.049**
Handgrip strength (kg)	20.1 ± 6.4	27.8 ± 7.8	**< 0.001**	20.4 ± 6.4	25.2 ± 8.3	**< 0.001**
Gait speed (m/s)	1.1 ± 0.3	1.2 ± 0.3	**< 0.001**	1.0 ± 0.3	1.2 ± 0.3	**< 0.001**
FTCS^6^ test (s)	14.0 ± 4.8	12.6 ± 4.2	**0.017**	14.3 ± 4.7	12.9 ± 4.4	**0.046**

^1^BMI, Body Mass Index; ^2^T2DM, Type 2 Diabetes Mellitus; ^3^uACR, urine albumin-to-creatinine ratio ^4^BMR, Basal Metabolic Rate; ^5^ASM, Appendicular Skeletal Muscle Mass; ^6^FTCS, Five-Times-Chair-Stand test

^†^Data from 112 T2DM insulin users (AWGS 2025: 57 sarcopenia, 55 non-sarcopenia; AWGS 2019: 19 sarcopenia, 93 non-sarcopenia)

**Fig. 3:**
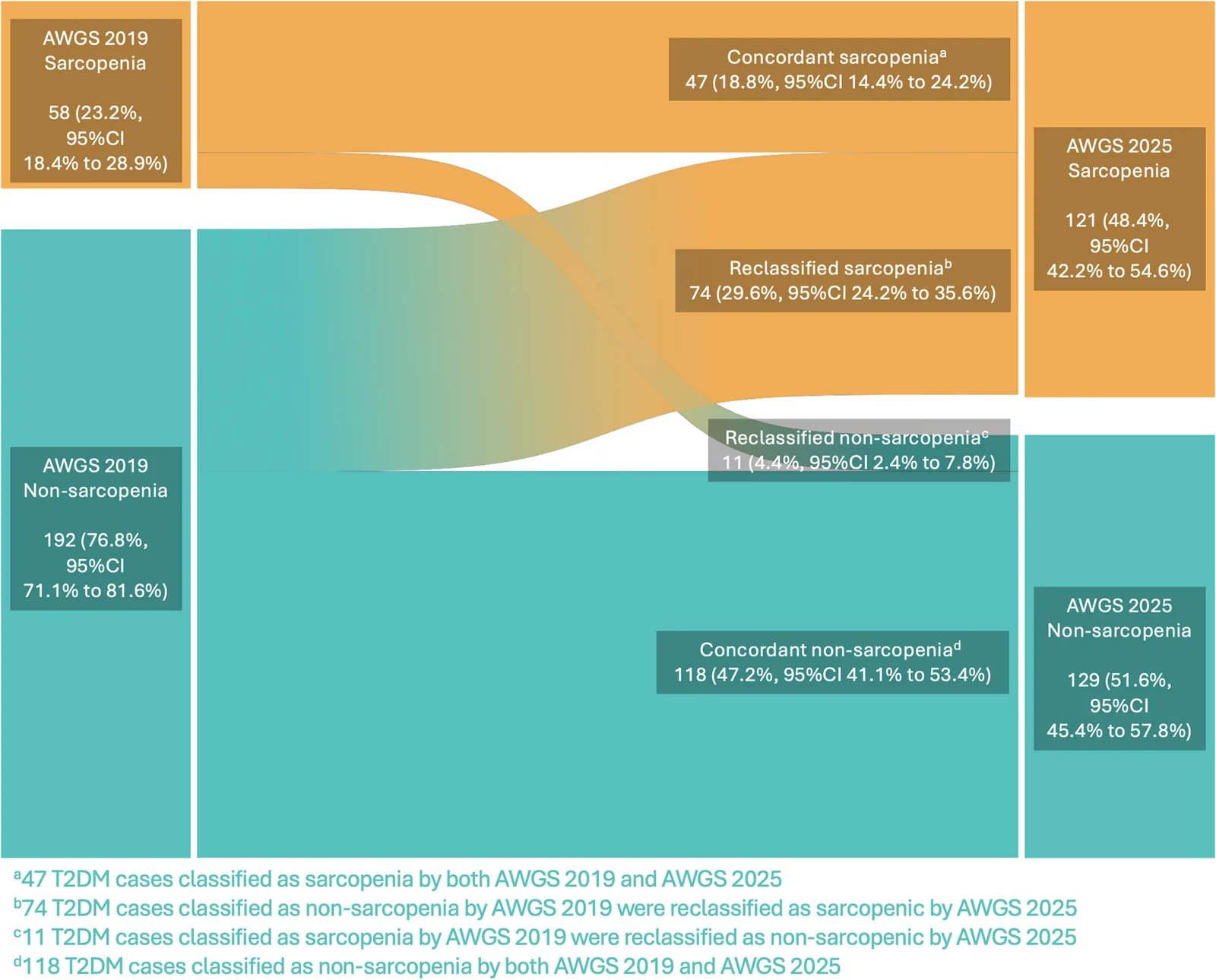
Diagram illustrating diagnostic transitions between AWGS 2019 and AWGS 2025 criteria

When examining diagnostic transitions between the two criteria, 11 patients (4.4%, 95%CI 2.4% to 7.8%) classified as having sarcopenia under AWGS 2019 were reclassified as non-sarcopenic under AWGS 2025. In contrast, 74 patients (29.6%, 95%CI 24.2% to 35.6%) previously identified as non-sarcopenic under AWGS 2019 were reclassified as sarcopenic by AWGS 2025 (Fig. 3). The remaining 47 patients (18.8%, 95%CI 14.4% to 24.2%) had concordant sarcopenia under both criteria, while 118 patients (47.2%, 95%CI 41.1% to 53.4%) were consistently classified as non-sarcopenic. Overall agreement between the two classifications was 66.0% (Cohen’s κ = 0.31). Of the 74 participants reclassified as having sarcopenia, 66 were reclassified solely by the BMI-adjusted ASM criterion, 6 by both the BMI-adjusted and revised height-adjusted ASM criteria, and 2 solely by the revised height-adjusted ASM criterion.

We then compared the characteristics of patients who were reclassified (reclassified sarcopenia and reclassified non-sarcopenia) with those whose diagnoses remained concordant under both criteria (concordant sarcopenia and concordant non-sarcopenia). Reclassified sarcopenic patients showed muscle strength and physical performance patterns similar to those with concordant sarcopenia, whereas their anthropometry and body composition more closely resembled the concordant non-sarcopenia group (Table 2). Conversely, reclassified non-sarcopenic patients (*n* = 11) retained handgrip strength and gait speed comparable to the concordant non-sarcopenic group, whereas their muscle mass, anthropometry, and body composition more closely resembled the concordant sarcopenic group. Among physical performance measures, only the five-times chair-stand test differed significantly, aligning with the concordant sarcopenic group. Given the small subgroup size, these comparisons should be interpreted with caution (Table 2).

**Table 2:** Characteristics and functional outcomes of concordant and reclassified diagnostic groups between AWGS 2019 and AWGS 2025 in patients with type 2 diabetes

Characteristics	AWGS 2025 Sarcopenia		AWGS 2025 Non-sarcopenia	
	Concordant sarcopenic^*^ n = 47	Reclassified sarcopenic^**^n = 74	Reclassified non-sarcopenic^***^ n = 11	Concordant non-sarcopenic^****^ n = 118
Age (years)	67.9 ± 9.0	65.2 ± 8.9	**71.5 ± 6.9** ^**b**^	63.4 ± 7.7
**Sex**,** n (%)**				
Male	25 (53.2%)	39 (52.7%)	6 (54.5%)	55 (46.6%)
Female	22 (46.8%)	35 (47.3%)	5 (45.5%)	63 (53.4%)
Weight (kg)	56.7 ± 9.1	**75.2 ± 12.6** ^**a**^	**59.8 ± 11.6** ^**b**^	76.4 ± 16.9
Height (cm)	155.3 ± 7.3	159.0 ± 6.8	158.8 ± 8.7	161.8 ± 8.2
BMI^1^ (kg/m²)	23.3 ± 3.7	**29.8 ± 4.6** ^**a**^	**23.7 ± 4.1** ^**b**^	29.0 ± 5.5
T2DM^2^ duration (years)	18.0 ± 10.9	16.3 ± 9.0	19.0 ± 9.8	14.7 ± 9.4
HbA1c (%)	7.8 ± 1.7	7.7 ± 1.4	7.4 ± 1.1	7.6 ± 1.6
**Insulin use**,** n (%)**				
No insulin therapy	30 (63.8%)	34 (46.0%)	9 (81.8%)	65 (55.1%)
Low (< 0.5 unit/kg/day)	13 (27.7%)	14 (18.9%)	2 (18.2%)	34 (28.8%)
Moderate (0.5-1.0 unit/kg/day)	4 (8.5%)	20 (27.0%)	0 (0.0%)	16 (13.6%)
High (> 1.0 unit/kg/day)	0 (0.0%)	6 (8.1%)	0 (0.0%)	3 (2.5%)
Total insulin use (unit/kg/day)†	0.39 ± 0.23	**0.62 ± 0.33** ^**a, b**^	0.13 ± 0.01	0.45 ± 0.28
**uACR**^**3**^, **n (%)**				
<30 mg/g	22 (46.8%)	35 (47.3%)	9 (81.8%)	75 (63.6%)
30–300 mg/g	17 (36.2%)	29 (39.2%)	2 (18.2%)	33 (28.0%)
>300 mg/g	8 (17.0%)	10 (13.5%)	0 (0.0%)	10 (8.5%)
BMR^4^ (kcal/day)	1208.0 ± 125.4	**1371.4 ± 157.6** ^**a**^	**1207.1 ± 119.7** ^**b**^	1437.8 ± 243.2
Body Fat (%)	30.6 ± 9.2	**37.7 ± 8.3** ^**a**^	33.8 ± 10.9	35.6 ± 8.7
ASM^5^ (kg)	14.3 ± 2.8	**18.7 ± 3.6** ^**a**^	**14.9 ± 3.3** ^**b**^	19.9 ± 4.7
ASM/ht² (kg/m²)	5.9 ± 0.7	**7.3 ± 1.0** ^**a**^	**5.8 ± 0.7** ^**b**^	7.5 ± 1.2
ASM/BMI	0.6 ± 0.1	**0.6 ± 0.2** ^**b**^	0.6 ± 0.2	0.7 ± 0.2
Handgrip strength (kg)	18.8 ± 5.5	**20.9 ± 6.9** ^**b**^	**27.1 ± 6.0** ^**a**^	27.8 ± 8.0
Gait speed (m/s)	1.0 ± 0.3	**1.1 ± 0.3** ^**b**^	1.1 ± 0.4	1.2 ± 0.2
FTCS^6^ test (s)	13.8 ± 4.6	**14.1 ± 4.9** ^**b**^	**16.6 ± 4.4** ^**b**^	12.2 ± 3.9

*T2DM patients classified as sarcopenic by both AWGS 2019 and AWGS 2025

**T2DM patients classified as non-sarcopenic by AWGS 2019 but sarcopenic by AWGS 2025

***T2DM patients classified as sarcopenic by AWGS 2019 but non-sarcopenic by AWGS 2025

****T2DM patients classified as non-sarcopenic by both AWGS 2019 and AWGS 2025

Bold values denote significance: ᵃ p<0.05 vs. concordant sarcopenic; ᵇ p<0.05 vs. concordant non-sarcopenic (continuous variables, one-way ANOVA with Bonferroni correction; categorical variables, Fisher exact test)

¹BMI, Body Mass Index; ²T2DM, Type 2 Diabetes Mellitus; ³uACR, urine albumin-to-creatinine ratio; ⁴BMR, Basal Metabolic Rate; ⁵ASM, Appendicular Skeletal Muscle Mass; ⁶FTCS, Five-Times Chair-Stand test

†Data from T2DM insulin users (concordant sarcopenic: 17, reclassified sarcopenic: 40, reclassified non-sarcopenic: 2, concordant non-sarcopenic: 53)

## Discussion

The AWGS 2025 criteria classified a substantially larger proportion of patients with T2DM as having sarcopenia than the AWGS 2019 definition. Participants additionally classified as sarcopenic under the AWGS 2025 criteria exhibited different metabolic characteristics, including higher BMI, greater body fat percentage, more frequent insulin use, and higher uACR, while showing broadly similar muscle-related characteristics to those classified as sarcopenic under the AWGS 2019 criteria. The clinical significance of these differences remains uncertain and requires prospective validation.

As expected, the prevalence of sarcopenia among patients with T2DM increased substantially under the updated criteria across most age groups. The apparent bimodal pattern, with peaks at 60–65 years and > 80 years, should be interpreted cautiously. This observation may reflect the age-specific diagnostic thresholds introduced in the AWGS 2025 criteria, particularly the transition at 65 years, rather than a true biological change in sarcopenia prevalence. In addition, the relatively small number of participants in some age strata, especially the oldest age group, may have contributed to fluctuations in the observed prevalence estimates. Because age-related declines in muscle strength and physical performance occur gradually rather than abruptly [22], larger studies are needed to determine whether this apparent pattern reflects a true epidemiological phenomenon or is attributable to the age-specific diagnostic thresholds and the limited number of participants in certain age subgroups.

Another explanation for the higher prevalence of sarcopenia among patients with T2DM under the AWGS 2025 criteria is the adoption of BMI-adjusted ASM [9]. By accounting for differences in body size, BMI-adjusted ASM may improve recognition of relative muscle deficiency, particularly in individuals with obesity or sarcopenic obesity, in whom height-adjusted ASM may underestimate impaired muscle health [23]. This modification may be especially relevant in T2DM because many patients have obesity, increased adiposity, and insulin resistance while maintaining relatively preserved absolute muscle mass. Consequently, muscle deficiency may be overlooked when muscle mass is assessed solely using height-adjusted ASM. Incorporating BMI into the calculation results in lower adjusted muscle mass values among individuals with higher BMI, making them more likely to meet the diagnostic threshold for sarcopenia. However, this approach also has important limitations. BMI cannot distinguish fat mass from lean mass. Therefore, BMI-adjusted ASM may increase the likelihood of overclassifying individuals with obesity who have preserved muscle reserve, potentially inflating sarcopenia prevalence in populations such as patients with T2DM [24]. Although BMI-adjusted ASM has been associated with functional decline and adverse clinical outcomes and was therefore incorporated into the FNIH and AWGS 2025 diagnostic criteria, whether these additional classifications represent clinically meaningful sarcopenia remains uncertain [9, 23, 25]. The substantial increase in sarcopenia prevalence under the AWGS 2025 criteria may increase the screening burden and healthcare resource utilization in diabetes clinics. Therefore, these findings should be interpreted with caution until their clinical implications are better established. Future prospective studies comparing different muscle mass indices against functional outcomes and other clinically relevant endpoints are needed to determine the optimal approach for diagnosing sarcopenia.

When comparing characteristics between sarcopenic and non-sarcopenic participants with T2DM under the two criteria, those classified as sarcopenic according to the AWGS 2025 definition tended to have higher insulin utilization and higher uACR levels. These descriptive findings are consistent with previous reports linking more advanced diabetes and diabetic kidney disease with muscle loss [5, 26]. The greater proportion of participants classified as sarcopenic among individuals with elevated BMI may further reflect the interaction between obesity, insulin resistance, and altered muscle metabolism. However, because this study was cross-sectional, it cannot determine whether these additional participants are at increased risk of adverse clinical outcomes or would derive greater benefit from targeted interventions. These observations therefore provide hypotheses for future prospective studies evaluating the clinical relevance of the revised AWGS 2025 criteria.

Assessment of diagnostic transitions between AWGS 2019 and AWGS 2025 showed that the updated criteria identified additional T2DM patients who were previously classified as non-sarcopenic under AWGS 2019 but demonstrated reduced muscle strength and impaired physical performance [9, 14]. These findings suggest that AWGS 2025 may broaden the identification of patients with reduced muscle health, despite the altered body composition characteristic of T2DM and the relatively high reclassification rate of nearly 30%. However, because only 11 participants were reclassified as non-sarcopenic, meaningful interpretation of this subgroup is limited. Although these participants exhibited borderline characteristics, with some measures appearing more similar to the concordant sarcopenia group and others resembling the concordant non-sarcopenia group, these observations should be considered hypothesis-generating rather than definitive. This finding highlights the uncertainty surrounding individuals with borderline phenotypes and underscores the need for further refinement and validation of diagnostic thresholds.

This study has several strengths. It is among the first to evaluate how sarcopenia prevalence changes under the newly proposed AWGS 2025 criteria in patients with T2DM. We also characterized the metabolic and functional features of patients identified by each definition, which may help improve awareness of sarcopenia in routine diabetes care. Nevertheless, several limitations should be acknowledged. First, six participants were younger than 50 years, which is outside the age range specified by the AWGS 2025 criteria. For these participants, the cutoffs for the 50–65-year age group were applied because no age-specific cutoffs are available for younger adults. However, sensitivity analysis excluding these participants yielded similar results, suggesting that this had minimal impact on the study findings. Second, most participants had relatively well-controlled T2DM, which may limit generalizability to patients with more severe disease. Third, although no participants were excluded because they were considered unsafe for physical performance testing, enrollment was limited to individuals who could safely complete the assessments. Consequently, patients with more severe functional impairment may be underrepresented, potentially limiting the generalizability of our findings. Fourth, the limitation relates to the use of BIA to estimate muscle mass. Although multi-frequency BIA is recommended by the AWGS and is practical for clinical use, its estimates may be influenced by hydration status, extracellular water distribution, edema, adiposity, and renal function. These factors are particularly relevant in patients with T2DM, who frequently have obesity and varying degrees of diabetic kidney disease. To minimize measurement variability, all BIA assessments were performed during a single morning visit under standardized conditions after overnight fasting, and participants with CKD stages 4–5 or other conditions likely to substantially alter body-water distribution were excluded. Nevertheless, residual measurement error cannot be completely excluded, and future studies using reference methods such as dual-energy X-ray absorptiometry (DXA) or magnetic resonance imaging (MRI) would be valuable to validate these findings. Fifth, although routine laboratory variables were obtained on the study visit date or within 7 days before assessment, selected non-routine laboratory tests (e.g., serum 25-hydroxyvitamin D) were obtained from the most recent available results within the preceding 3 months when repeat testing was not clinically indicated. Although these variables were not primary analytical variables, some measurement heterogeneity cannot be completely excluded. Sixth, the sample size was relatively small, limiting the ability to perform further subgroup analyses. In particular, the reclassified non-sarcopenia subgroup comprised only 11 participants, limiting the precision of subgroup comparisons and precluding firm conclusions regarding their clinical characteristics. Finally, this study was a secondary analysis of a cross-sectional dataset, which limits the interpretation of our findings to differences in participant classification and characteristics between the AWGS 2019 and AWGS 2025 criteria. The study cannot determine whether the AWGS 2025 criteria are more accurate in predicting future clinical outcomes. Prospective longitudinal studies are needed to compare the predictive performance of the two diagnostic criteria for clinically relevant outcomes, including functional decline, disability, falls, fractures, hospitalization, and mortality.

## Conclusions

Application of the AWGS 2025 criteria substantially increased the prevalence of sarcopenia and altered diagnostic classification among patients with T2DM compared with the AWGS 2019 criteria. In unadjusted analyses, participants additionally classified as sarcopenic exhibited different metabolic characteristics, including higher BMI, greater insulin utilization, and elevated uACR. However, these findings were based on cross-sectional descriptive analyses, and the clinical significance of these diagnostic reclassifications remains uncertain. Prospective longitudinal studies are needed to determine whether the revised criteria improve the prediction of clinically relevant outcomes.

## Supplementary Information

Below is the link to the electronic supplementary material.

Supplementary Material 1

## Data Availability

The datasets generated and/or analyzed in this study are not publicly available due to institutional and ethical restrictions. However, the data are available from the corresponding author upon reasonable request and with permission from the relevant institution.

## Abbreviations

AGEs: Advanced glycation end products
ASM: Appendicular skeletal muscle mass
AWGS: Asian Working Group for Sarcopenia
BIA: Bioelectrical impedance analysis
BMI: Body mass index
BMR: Basal metabolic rate
EWGSOP: European Working Group on Sarcopenia in Older People
FNIH: Foundation for the National Institutes of Health
FTCS: Five-times chair stand
HbA1c: Glycated hemoglobin
T2DM: Type 2 diabetes mellitus
uACR: Urine albumin-to-creatinine ratio
