# Supplementary material for: Prevalence and diagnostic reclassification of sarcopenia in type 2 diabetes according to AWGS 2019 and AWGS 2025 criteria: a secondary cross-sectional analysis
Source: BMC Endocr Disord. 2026 Aug 13;26:365. doi: 10.1186/s12902-026-02428-2 (PMC13753356; doi:10.1186/s12902-026-02428-2)
Supplement: Supplementary file 1 [file 12902_2026_2428_MOESM1_ESM.docx]

**Supplementary table**

**Supplementary Table 1.** Definition of sarcopenia according to Asian Working Group for Sarcopenia (AWGS) 2025 and 2019

| **Diagnosis criteria** | **AWGS 2025** | | **AWGS 2019** |
| --- | --- | --- | --- |
|  | Age 50-64 years | Age ≥64 years |  |
| **Appendicular skeletal muscle mass (ASM)** | | | |
| Bioelectrical impedance analysis |  |  |  |
| Height-adjusted (kg/m^-2^) | M:<7.6, F: <5.7 | M:<7.2, F: <5.7 | M:7.0, F: <5.7 |
| BMI-adjusted | M:<0.90, F: <0.63 | M:<0.83, F: <0.57 |  |
| *and* | | | |
| **Muscle function evaluation** | | | |
| 1. Muscle strength |  |  |  |
| Hand grip strength (kg) | M: <34, F <20 | M: <28, F <18 | M: <28, F <18 |
| *or* | | | |
| 2. Physical performance |  | |  |
| Gait speed (m/s) |  |  | <1.0 |
| 5-time chair stand test (s) |  |  | ≥12 |
